# RNA N^6^-methyladenosine modification in female reproductive biology and pathophysiology

**DOI:** 10.1186/s12964-023-01078-4

**Published:** 2023-03-09

**Authors:** Erqing Huang, Lijuan Chen

**Affiliations:** Department of Obstetrics and Gynecology, Union Hospital, Tongji Medical College, Huazhong University of Science and Technology, Wuhan, 430022 China

**Keywords:** m^6^A methylation, Epigenetic modification, Reproductive disease, Preeclampsia, Endometriosis, Abortion, Gynaecological cancer

## Abstract

Gene expression and posttranscriptional regulation can be strongly influenced by epigenetic modifications. N^6^-methyladenosine, the most extensive RNA modification, has been revealed to participate in many human diseases. Recently, the role of RNA epigenetic modifications in the pathophysiological mechanism of female reproductive diseases has been intensively studied. RNA m^6^A modification is involved in oogenesis, embryonic growth, and foetal development, as well as preeclampsia, miscarriage, endometriosis and adenomyosis, polycystic ovary syndrome, premature ovarian failure, and common gynaecological tumours such as cervical cancer, endometrial cancer, and ovarian cancer. In this review, we provide a summary of the research results of m^6^A on the female reproductive biology and pathophysiology in recent years and aim to discuss future research directions and clinical applications of m^6^A-related targets. Hopefully, this review will add to our understanding of the cellular mechanisms, diagnostic biomarkers, and underlying therapeutic strategies of female reproductive system diseases.

Video Abstract

**Supplementary Information:**

The online version contains supplementary material available at 10.1186/s12964-023-01078-4.

## Background

As a result of the growing interest in understanding how DNA and RNA modifications function, epigenomics has become a cutting-edge field [1]. Scientists have identified numerous layers of epigenetic modulation that are derived from the modification of DNA and proteins; however, RNA modification remains elusive [2]. Over 160 RNA chemical modifications have been discovered thus far [3]. The multitude of RNA modifications has added a new level of complexity to gene regulation. In eukaryotic mRNA, m^6^A RNA modification is the most abundant modification, which is defined as the methylation of adenosine 6 positions in mRNA and some noncoding RNA. N^6^-methyladenosine has been proven to be connected with various metabolic and physiologic processes, such as RNA transcript splicing, translation efficiency, nuclear export, stability, and decay [4]. m^6^A shares the same base pairing with unmodified adenosine, which prevents it from being detected by conventional sequencing or hybridization methods. A mystery surrounded the transcriptome distribution of m^6^A until a combination of m^6^A-specific methylated RNA immunoprecipitation and next-generation sequencing was devised. By employing these methods, scientists were able to determine that m^6^A residues were located in evolutionarily conserved regions within humans and mice. m^6^A residues were found to be contained primarily within internal mRNA sequences and appeared in the poly A tail, especially in the 3'UTR of mRNAs near the stop codon, and they always share a consensus motif of RRACH (R = A/G, H = A/C/U), and a terminal U is dominant part of the consensus [5–8].

The process of RNA methylation is controlled by methyltransferases, demethylases, and recognition factors. A methyltransferase and a demethylase catalyse the methylation of RNA dynamically, and the m^6^A function is determined by its recognition factor. Regulators may also be referred to as ''writers'', ''erasers'', and ''readers''. Although these regulators have been identified, the specific function of this process has not been well established. Cell types and environmental conditions may affect the expression level and biological function of m^6^A effectors. Furthermore, these factors can also be influenced by RNA species, abundances, secondary structures, intracellular location, translation state, and other heterogeneous factors. Taking these factors into account, it may be difficult to explain the exact function of m^6^A [9].

The normal physiological functions of the female reproductive system depend on the gonadal axis of the reproductive endocrine system, which regulates ovulation and the menstrual cycle. The maintenance of normal pregnancy depends on the establishment of endometrial receptivity, normal embryogenesis, and the formation of an immune microenvironment at the maternal-foetal interface. Several studies have shown that m^6^A modification is related to the regulation of physiological functions of the female reproductive system [10]. For example, by controlling the translation of Pgr mRNA through m^6^A modification, METTL3 is crucial for efficient P4 signaling during embryogenesis. m^6^A‑RNA immunoprecipitation‑qPCR showed that Pgr mRNA transcript was a target for METTL3-dependent m^6^A mRNA methylation [11]. In the process of ovarian aging, researchers found that the ovarian aging can be alleviated by up-regulating the FTO level in ovarian granulosa cells to reduce the m^6^A level of FOS-mRNA-3′UTR [12].However, the subtle mechanism of m^6^A modification involved in the physiological function of the female reproductive system remains to be elucidated.

Common disorders of the female reproductive system include benign diseases, such as inflammatory diseases of the reproductive organs, infertility, endometriosis, and polycystic ovarian syndrome; complications of pregnancy, such as miscarriage, preeclampsia, and gestational diabetes mellitus; and some malignant diseases, such as cervical cancer, endometrial cancer, and ovarian cancer. In recent years, it has been found that epigenetic abnormalities may be associated with the development of several female reproductive disorders. For example, epigenetic modifications such as DNA methylation, histone methylation and acetylation and long noncoding RNAs are involved in regulating follicular granulosa cell proliferation, endometrial receptivity and decidualization in early pregnancy, which affect ovulation, embryo implantation, and labour initiation [13]. Abnormalities in these epigenetic modifications may be associated with polycystic ovary syndrome, infertility due to repeated embryo implantation failure, and pregnancy complications. Some patients with endometriosis may be born with congenital epigenetic molecular abnormalities, and the recurrent bleeding and repair processes of endometriosis lesions may also trigger acquired epigenetic events [14]. In addition, some chemical toxicants, such as Bisphenol A (BPA), can affect ovarian function, embryonic development, and gamete quality during fertilization through epigenetic modifications such as CpG island methylation, histone modifications, and noncoding RNA production [15].

The function of epigenetic modification in the female reproductive system has received increasing attention over the last few years. Despite this, research on m^6^A modification and the female reproductive system is still in its infancy. A comprehensive review of recent research advances in m^6^A modification and its roles in pathogenesis, diagnosis, and molecular targeted therapies for gynaecological reproductive disease is presented in this paper. We discuss the potential directions of m^6^A modification for future research in obstetrical and gynaecological disorders, aiming to clarify the role played by RNA methylation in female reproductive system diseases and malignant tumours. In doing so, we can gain a novel perspective on these diseases.

## The mechanism and regulation of m^6^A RNA methylation

### m^6^A writers

**Fig. 1:**
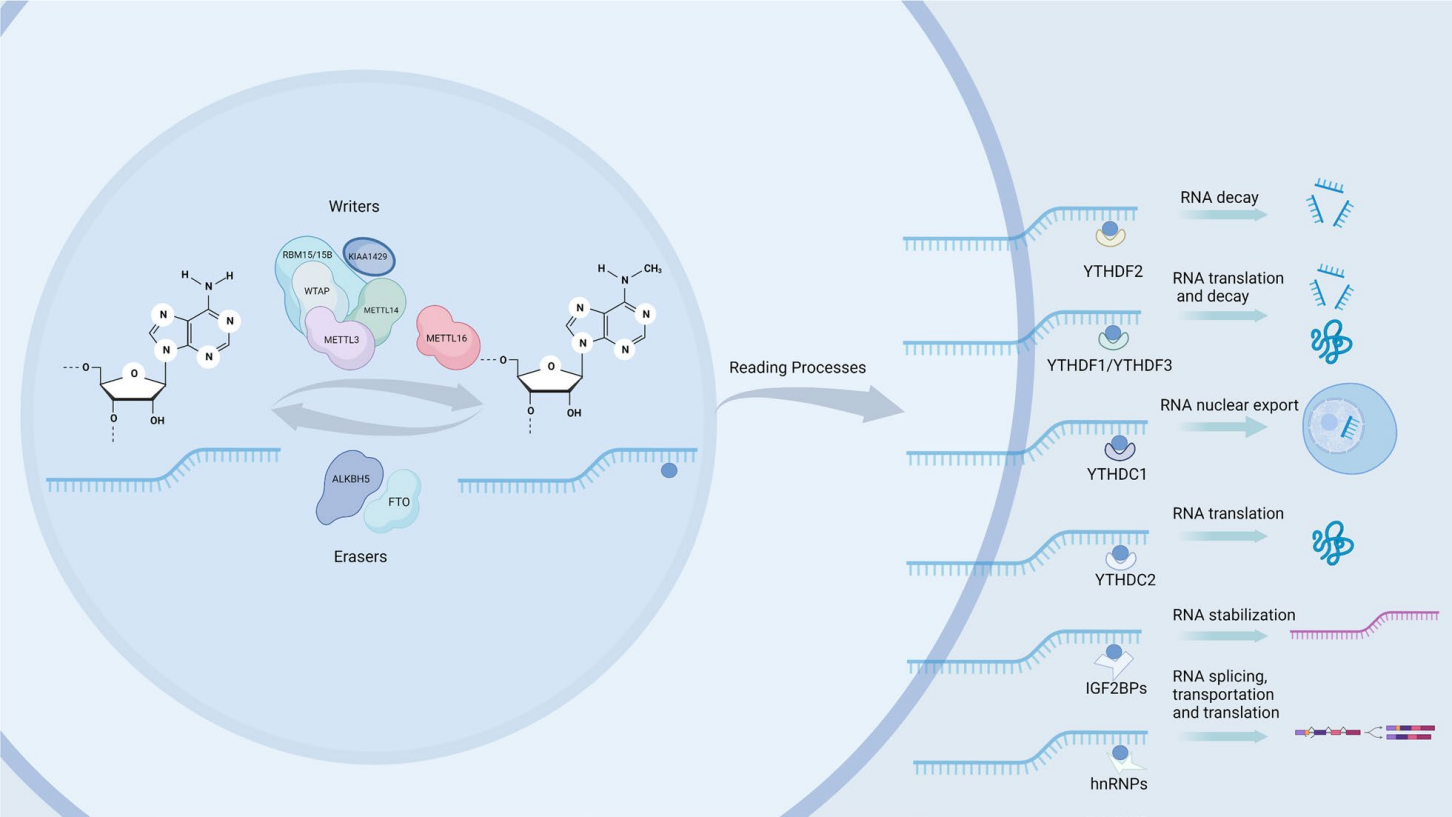
Detailed mechanism of m^6^A readers, writers, and erasers. N^6^-methyladenosine is methylated by the methyltransferase complex, which contains METTL3, METTL14, WTAP, KIAA1429, and RBM15/15B. The m^6^A erasers FTO and ALKBH5 can reversibly remove m^6^A. The reader is responsible for performing the biological functions of m^6^A, such as RNA translation, decay, splicing, and translation

An N^6^-adenosine methyltransferase complex (MTC) is required for the installation of this modification (Fig. 1). The MTC comprises the METTL3 catalytic subunit, as well as the METTL14, WTAP, VIRMA, RBM15, and ZC3H13 accessory subunits. A 70 kDa protein named METTL3 was the first known m^6^A writer [3, 16]. The METTL3 subunit is a key component of the catalytic process, facilitating the reception of methyl groups from the SAM moiety by RNA adenine. METTL14, a molecular homologue of METTL3, was discovered by researchers as a new methyltransferase [17]. When METTL14 and METTL3 combine, a stable heterodimer is formed. It is believed that this complex mediates m^6^A deposition on nuclear RNA in mammals. A structural requirement for the activation of METTL3 is METTL14. METTL14 acts as an essential component to facilitate RNA recognition and binding. Although METTL14 is not directly involved in catalysis, it stabilizes the conformation of METTL3 to enhance catalytic activity [18, 19]. By decreasing METTL14 expression, methylated RNA binding and the splicing-related protein DGCR8, which plays an important role in maturing RNA, were suppressed [20].

RNA-binding domains in RBM15 and RBM15B enable WTAP–METTL3 to bind to specific mRNAs and determine the methylated m^6^A consensus sequences. As part of the m^6^A methylation complex, RBM15/15B binds to XIST, and X-chromosome genes can be silenced by this transcription factor [21]. Researchers have identified that WTAP contains no obvious catalytic domains, but it initiates the localization of METTL3-METTL14 in the nuclear speckle and facilitates catalysis. Both WTAP and METTL3 regulate transcription and RNA processing genes by regulating their expression and alternative splicing. Morpholino-mediated knockdown of WTAP or METTL3 in zebrafish embryos resulted in defects in tissue differentiation and an increase in apoptosis [22].

Interestingly, it has been reported that WTAP-dependent methylation is inversely correlated with mRNA stability and appears to be depleted in housekeeping genes; highly plentiful messages may have evolved to avoid mRNA methylation, thereby optimizing their stability [23]. In HeLa cells, KIAA1429 mediates the preferred deposition of m^6^A in the 3'UTR near the stop codon and contributes to APA [24]. Furthermore, its homologue, Virilizer, is essential for the alternative splicing of the sex determination factor in *Drosophila *[25].

Due to METTL16’s role in regulating SAM homeostasis, it has been linked to several modifications in the epitranscriptome of m^6^A [26]. Scientists have investigated whether METTL16 methylates MAT2A (encoding SAM synthetase) and U6 snRNA [27]. METTL16 has been found to influence early embryonic development through the regulation of SAM synthetase expression in mouse studies [28]. Homologues of METTL16 can be found in a wide range of organisms; however, only the MAT2A hairpin and two carboxyl-terminal VCRs of METTL16 are conserved among vertebrates. Researchers have demonstrated that splicing is controlled by the METTL16 VCRs, whereas transcription is controlled by the methyltransferase domain. It appears that SAM levels are sensitive to METTL16-dependent m^6^A sites. In addition, it appears that SAM is generated by splicing the retained MAT2A intron under the control of METTL16 occupancy at hp1 under SAM-limiting conditions [27, 29, 30].

### m^6^A readers

A significant proportion of the effects of m^6^A on targeted mRNAs are related to the functions of various mRNA readers, which are proteins that bind to m^6^A. These include members of the YT521-B homology domain (YTH) family, heterogeneous nuclear ribonucleoproteins (HNRNPs) and insulin-like growth factor 2 mRNA-binding proteins (IGF2BPs). Furthermore, m^6^A readers are involved in several biological processes in addition to participating in RNA metabolism [31]. YTH proteins recognize m^6^A residues through three of the YTHDF domain families (DF1, DF2, and DF3), YTHDC1 (DC1), and YTHDC2 (DC2). The RNA-binding domain of YTH is highly conserved in eukaryotes [32]. Although all five human YTH domain-containing proteins contain homologous YTH domains, their biological functions were unknown until YTHDF2 demonstrated that recognizing m^6^A through its YTH domain affects the lifespan of mammalian mRNAs. Over 3,000 cellular RNA targets of YTHDF2 have the conserved core motif of Gm^6^AC [33]. Deadenylation and decay of m^6^A-containing mRNA are initiated by YTHDF2 by recruiting CNOT1 to the CCR4-NOT complex [34]. YTHDF3 and YTHDF1 cooperate to promote translation and enhance RNA decay. In addition, YTHDF3 can strengthen RNA decay mediated by YTHDF2 [35]. In association with SRSF3, YTHDC1 recognizes methylated mRNA and delivers it to the nuclear mRNA export receptor NXF1 [36]. Researchers surmised that YTHDC2 plays an important role in increasing translation efficiency and inhibiting mRNA stability through its binding of m^6^A and its translation and decay machinery [37]. An analysis of CDS m^6^A modification demonstrated that CDS methylation regulates translation by resolving the secondary structure of mRNA. Only one RNA helicase, YTHDC2, contains the m^6^A reader, which is required for this translation-promoting effect of m^6^A modification [38].

A family of single-stranded RNA binding proteins (RBPs) known as IGF2BPs consists of six canonical RNA binding domains, including two RNA recognition motif (RRM) domains and four K homology (KH1-4) domains [39]. IGF2BPs recognize m^6^A via their K homology domains. However, the IGF2BP2 protein appears to be the only member of this family that regulates the fate of mRNA in non-transformed adult tissues, suggesting that it may be involved in metabolic regulation [40]. Stabilization of targets is achieved by the recruitment of RNA stabilizers by IGF2BPs. Moreover, IGF2BPs colocalized well with stress granule markers, messenger ribonuclear protein granules in the cytoplasm that are thought to protect mRNA from harmful conditions so that it can be translated once the stress is relieved. Interestingly, IGF2BP1 was also shown to stabilize mRNA under stress. In tissues suffering from heat shock and recovering from it, IGF2BPs colocalize with stress granules and shuttle between ribosomal and nonribosomal fractions, suggesting that they play a role in mRNA translation during the stress response [41]. MYC has been proven to be a target of IGF2BP1, and the reader binds MYC through a cis-acting element located within its 3' terminus called the coding region instability determinant (CRD) [42].

The HNRNPS family is composed of HNRNPC, HNRNPG, and HNRNPA2B1. HNRNPG interacts with RNA polymerase II to regulate alternative splicing of m^6^A-modified nascent RNAs [43]. The local structure of m^6^A-modified mRNA or lncRNA is changed, making it easier to bind to HNRNPC. HNRNPC participates in the alternative splicing, transportation, and translation of pre-RNA [44]. HNRNPA2B1 was identified as an m^6^A reader since it can also promote the alternative splicing of exons with METTL3. In addition, HNRNPA2B1’s interaction with the DGCR8 protein endows it with the ability to process pre-miRNAs [45].

### m^6^A erasers

In previous studies, the fat mass and obesity-associated gene (FTO) has been shown to be related to childhood obesity and type 2 diabetes (T2D). Further research revealed that FTO is associated not only with increased body mass index (BMI) but also with m^6^A demethylase activity [46]. Cellular mRNA contains less m^6^A due to the oxidation activity of FTO [47]. α-Ketoglutaric acid and Fe(II) are required for the oxidative demethylation of m^6^A by FTO. On the inhibition of nucleolar RNA polymerase II transcription, a pronounced concentration of FTO in specks was observed, suggesting that FTO interacts dynamically with nuclear specks [48]. Multiple mRNA metabolism factors are associated with splicing speckles [49]. It is possible that FTO contributes to the splicing process. FTO has also been found to play a new role in alternative splicing. m^6^A and FTO are thought to play a role in poly(A) site selection and possible 3'UTR length regulation. Notably, the demethylation of RNAs by FTO is not sequence specific, and FTO has significantly higher catalytic activity for demethylating m^6^Am than for m^6^A [40, 48, 50–52].

ALKBH5 is also a member of the AlkB dioxygenase family. ALKBH5 has emerged as the second known RNA demethylase in mammals. In addition to oxidatively reversing m^6^A, researchers have discovered that this activity may affect RNA metabolism, mRNA export, and the assembly of nuclear speckle processing factors. There is evidence that ALKBH5 is localized to the nucleus, and its activity is dependent on 2OG and iron. ALKBH5's active site is more open than that of FTO, potentially because FTO has a longer NRL and a C-terminal domain, both of which function to enclose the active site. ALKBH5 and FTO, both m^6^A demethylases containing a basic residue, Lys132, and Arg96 in their active sites, may be involved in substrate selection or the release of the final product after demethylation [53].

## The physiological function of m^6^A RNA methylation in the female reproductive system

### Germ cell development

Cell proliferation, differentiation, and metabolism and the cell cycle in the female reproductive system are regulated by m^6^A factors, according to many studies. Maternal factors accumulate in oocytes and play a major role in embryo maturation and development. Transcriptional activity is silenced during this time from fully developed GV oocytes to mid-blastula embryos [54]. In *X. laevis* oocyte maturation and later embryo development, RNA methylation has been found to be responsible for RNA translation regulation [55]. Oocyte maturation requires precise control over translation due to the lack of transcription during the early stages of embryogenesis [56]. High methylation levels of maternal RNA can interfere with translation and reduce the amount of protein available for embryo development and oocyte maturation. Postnatal oocyte maturation is influenced by YTHDC1. Knocking down YTHDC1 post-natally induces female mice to lose their sterility and ability to produce secondary and antral follicles. YTHDC1 deficiency led to RNA metabolism defects and granule accumulation in oocytes. Moreover, loss of YTHDC1 is lethal to embryos. To be precise, when the m^6^A reader YTHDC1 is lost, the 3'UTR length is changed by extensive alternative polyadenylation. microRNAs and protein binding sites have been demonstrated to be abundant in the 3'UTR. In the absence of YTHDC1, transcription and translation are affected, and therefore, oocyte development and female fertility are compromised [57].

Mice with YTHDC2 deficiency survived. However, mice of both sexes lacked fertility, and female mice had smaller ovaries. No germ cells were able to progress beyond the zygotene stage of prophase I of meiosis. Through its binding to m^6^A, YTHDC2 increases translation efficiency and stabilizes its targets [37]. YTHDC2 in the testes of male mice can ameliorate Mn-induced reproductive toxicity as well as cell cycle arrest [58]. YTHDF1 knockdown inhibited the self-renewal of female mouse germline stem cells. A significant difference was found in total m^6^A levels and METTL3, ALKBH5, YTHDF1, YTHDF2, YTHDC1, and YTHDC2 in female mouse germline stem cells compared to Sandos inbred mouse embryo-derived thioguanine and ouabain-resistant cells [59]. The m^6^A reader YTHDF2 post-transcriptionally regulates transcript degradation during mouse oocyte meiosis maturation, which is required for early embryo development. YTHDF2-deficient mice can survive normally; however, female mice are infertile [60]. Several m^6^A readers, including IGF2BP2 and IGF2BP3, are implicated in stabilizing maternal mRNAs involved in DNA repair and meiosis during oogenesis. In addition to contributing to mRNA stability, m^6^A is potentially involved in oocyte maturation through its involvement in other RNA metabolism processes [61].

A high level of METTL3 expression was observed during follicle development and oocyte maturation. Oocytes with inactivated METTL3 develop improperly, produce abnormal follicles, and fail to ovulate correctly. METTL3 interacts with ITSN2, a protein involved in oocyte meiotic resumption, to enhance its stability to act on oocyte meiosis in an m^6^A-dependent manner. MII oocytes and offspring are not produced in METTL3 knockdown females, and oocytes in the GV stage have smaller numbers and diameters than wild-type oocytes, suggesting that METTL3 is an m^6^A methyltransferase that promotes oocyte development by stabilizing maternal mRNAs during the GV stage [61]. Knockdown of METTL3 did not affect meiotic resumption. However, METTL3-deficient oocytes were unable to produce sufficient first polar bodies and normal spindles [62].

### Embryo development

Many previous studies have confirmed that m^6^A regulators such as METTL3, METTL14, ALKBH5, and YTHDF2 regulate the differentiation of bone marrow mesenchymal stem cells, haematopoietic stem cells, and liver and breast cancer stem cells through Smad signalling, Notch signalling, and the oncogenes MYC and MYB [63–72]. Similarly, during embryonic development, the loss of METTL3 increases the expression of naïve pluripotency transcripts in murine pluripotent embryonic stem cells, hampering priming and differentiation competence [73]. IGF2BP3 binds to maternal mRNAs and inhibits their degradation during the transition between maternal and zygotic stages. Deficiency of IGFR2BP3 speeds up the degradation of maternal RNA. Cell division was impaired in IGF2BP3-mutant cells, but oocyte development was normal [74]. An in vitro experiment confirmed that deleting maternal IGF2BP2 causes interrupted embryonic development at the two-cell stage and impaired fertility. Oogenesis was not affected by IGF2BP2 deletion. In two-cell stage embryos, transcriptional and translational activity are both decreased. IGF2BP2 can significantly strengthen the ability of embryos to develop into blastocysts and improve embryo quality by regulating IGF2 [75].

In addition to mRNA methylation, in recent years, it has been reported that rRNA methylation also plays an important regulatory role in embryonic differentiation. Zebrafish that lose snoRNA-guided rRNA modification undergo developmental defects and die during embryogenesis [76]. Likewise, there is evidence that METTL5 methylates 18S rRNA. By deleting METTL5, the pluripotency of mouse embryonic stem cells (mESCs) is lost, and germ layer specification is impaired. Mechanistically, METTL5-deposited m^6^A promotes the translation of FBXW7, which plays a significant role in regulating cell differentiation. The absence of METTL5 delays the onset of mESC differentiation by inducing abnormal translation of FBXW7 and accumulation of its substrate, c-MYC [77]. All these studies indicate that m^6^A modification in mammals regulates germ cell differentiation by balancing the overall levels of m^6^A regulators, providing new insight into the functions and regulatory mechanisms of oocyte development.

### Foetal growth

In addition to being involved in the regulation of female germ cell development and embryonic development, the growth of the foetus has been shown to be affected by the m^6^A demethylase FTO. Placenta from newborns with a large head circumference or heavy birth weight is reported to express higher levels of the m^6^A demethylase FTO. This is consistent with the research about birth weight mentioned below, which showed that samples from heavy-for-date newborns had conspicuously reduced methylation. Maternal parity influences the expression level of the FTO gene in the human term placenta and its association with foetal and placental weights. In comparison to appropriate-for-date children, both the small birth weight and heavy birth weight groups showed high levels of m^6^A modifications at the 5'UTR but lower levels near stop codons [78, 79]. In low-birth-weight placentas of pigs with maternal obesity, FTO protein levels and m^6^A modification of FTO mRNA are higher. In addition, in low-birth-weight foetuses, genes related to lipid metabolism and angiogenesis are expressed differently than in normal foetuses. It is thought that increased m^6^A modification may play a role in regulating the expression of key genes in the low-birth-weight placenta [80]. Although the exact mechanism by which FTO impacts the head size of the foetus remains unclear, we presume that FTO may regulate foetal growth by m^6^A methylation [81].

Previous studies have shown that DNA methylation may be involved in the development of metabolic diseases in offspring through complex epigenetic effects. In a study of the risk of developing obesity in rodent offspring, scientists found that the expression of METTL3 and FTO genes was higher in the offspring of parents who ate a low-fat diet despite hypothalamic m^6^A modification levels being reduced. Notably, a ubiquitously expressed transcriptional coactivator, CREBBP, has elevated m^6^A modifications and increased mRNA expression in the offspring hypothalamus. CREBBP is known to be involved in the regulation of the body's nutritional status and energy balance by the hypothalamus. Therefore, it is possible to understand why low-fat parental diets result in a higher level of weight and insulin resistance in their offspring [82]. Table 1 summarizes the physiological functions of m^6^A RNA methylation in the female reproductive system.

**Table 1:** The physiological function of m6A RNA methylation in female reproductive system

Physiological function	m^6^A regulator	Type	Mechanism	References
Germ cell development	YTHDC1	Reader	Change 3'UTR length by extensive alternative polyadenylation	[57]
	YTHDC2	Reader	Influence translation efficiency and stabilize its targets	[37]
	YTHDF1	Reader	Associated with self-renewal of female mouse germline stem cells	[59]
	YTHDF2	Reader	Post-transcriptionally regulate transcript degradation during mouse oocyte meiosis	[60]
	METTL3	Writer	Interact with Itsn2 to act on oocyte meiosis in an m^6^A-dependent manner and involved in producing first polar bodies and normal spindles	[61, 62]
	IGF2BP2/IGF2BP3	Reader	Stabilize maternal mRNAs involved in DNA repair and meiosis during oogenesis	[61]
Embryo development	METTL3	Writer	Associated with the expression of naïve pluripotency transcripts	[73]
	IGF2BP3	Reader	Deficiency speeds up the degradation of maternal RNA	[74]
	IGF2BP2	Reader	Strengthen the ability of embryos to develop into blastocysts	[75]
	METTL5	Writer	Improve pluripotency of mouse embryonic stem cells and promote germ layer specification	[77]
Foetal growth	FTO	Eraser	Regulate the genes that control nutrient metabolism	[79, 82]

## m^6^A RNA methylation in pregnancy and female reproductive disorder

### Preeclampsia and gestational diabetes mellitus

Research has found that placental trophoblasts of preeclampsia patients had significantly higher levels of m^6^A, METTL3, and HNRNPC1/C2 [83]. A recent study demonstrated that METTL3 promotes m^6^A RNA methylation in the placenta of preeclamptic patients, resulting in a change from pre-miRNA 497-5p/145-5p to mature miRNA 497-5p/145-5p [84]. In preeclampsia samples, the levels of several heat shock proteins were elevated, and the peak of m^6^A was primarily located in the CDS region near the 3'UTR. Researchers have discovered that the m^6^A readers IGF2BPs can enhance gene expression by stabilizing mRNA when bound to mRNA [31, 85]. IGFBP2 binding sites are largely located in CDS regions near the stop codons and 3'UTRs. Based on these results, it is logical to assume that m^6^A modification increases the stability of HSPA1, which promotes its expression and plays a role in the pathogenesis of preeclampsia [86]. The 5'UTR m^6^A modification levels may provide foetal growth information that RNA expression analysis alone is unable to offer due to its variation. In addition, their analysis indicated that specific m^6^As were present in the 5'-UTRs of each foetal growth category. Furthermore, preeclampsia placentas from small-for-date children exhibited unique gene expression patterns not found in other placentas. They showed that 5'UTR methylation increased SMPD1 protein levels in the placenta of women with small-for-date children and preeclampsia through regulation of mRNA translation instead of elevating mRNA levels [78].

Recent studies have reported that circRNA methylation is related to the pathogenesis of preeclampsia. Specifically, the m^6^A reader IGF2BP3 increases the stability of circRNA PAPPA2 as a result of its interaction with m^6^A. IGF2BP3 and circRNA PAPPA2 levels are decreased in preeclamptic placentas compared with normal placental tissue, further impairing trophoblast cell invasion and leading to abnormal uterine spiral artery remodelling, which is a key pathogenic determinant of hypertension in pregnancy [87]. Studies have confirmed that RNA methylation and histone methylation can jointly participate in the pathogenesis of preeclampsia. Mechanistically, the RNA demethylase ALKBH5 was significantly upregulated in the preeclamptic placenta, which reduced the stability of PPARG mRNA and hindered its translation. Reduced PPARG inhibited the histone demethylase KDM3B from demethylating ALCAM, thereby reducing ALCAM expression, which eventually leads to preeclampsia [88]. METTL14 was found to be reduced in the placentas of mothers with gestational diabetes mellitus, which in turn decreased BAMBI m^6^A levels, thereby causing a lower BAMBI expression level. It was hypothesized that BAMBI reduction causes insulin resistance and impaired insulin sensitivity in islet β cells through effects on TGF-β and Wnt signalling, increasing the risk of gestational diabetes mellitus [89].

### Abortion

**Fig. 2:**
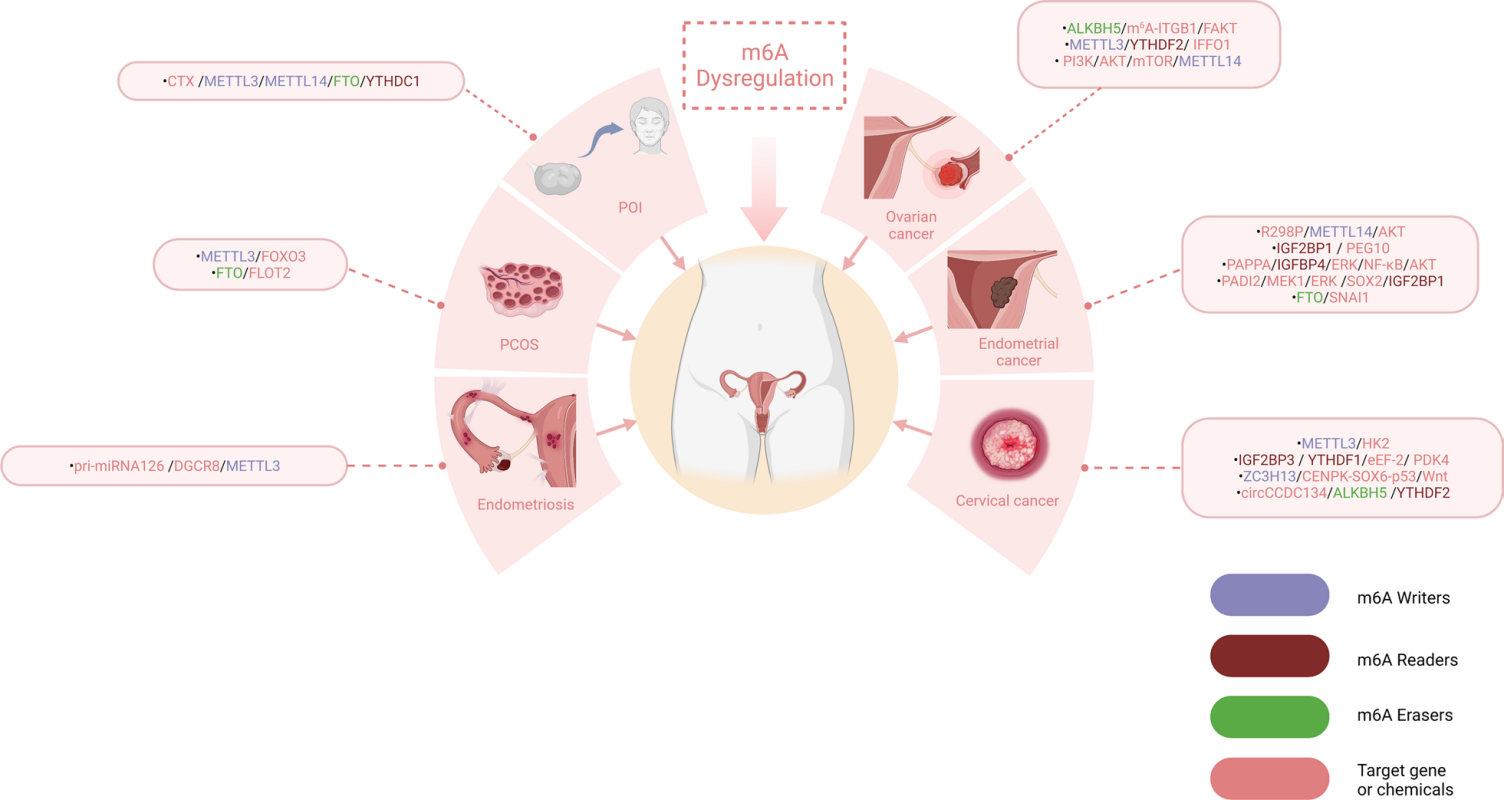
The functions of m^6^A regulators in female reproductive disorder. m^6^A regulators play a critical role in the pathological processes of the female reproductive system. By modulating different pathways and downstream targets, they influence the pathogenesis of female reproductive disorders

Properly controlled invasion of extravillous trophoblasts is crucial to enabling healthy development of the placenta in the uterus. Pregnancy-related complications, such as abortion and preeclampsia, can arise from abnormal growth and differentiation of trophoblast cells. Spontaneous abortion is the most common complication of pregnancy. Trophoblast cells exert an essential role in placental development. During pregnancy, trophoblastic molecule dysfunction can result in severe complications [90–92]. METTL14 is elevated in human trophoblast cells exposed to BPDE and in villous tissue from women suffering from recurrent miscarriages. METTL14 increases the m^6^A modification level of lnc-HZ01, enhancing RNA stability and participating in a loop of self-feedback of MXD1/METTL14/lnc-HZ01. Abnormal activation of this loop hinders the proliferation of trophoblast cells [93]. The RNA demethylase ALKBH5 was explicitly elevated in placental villous tissue from recurrent miscarriage patients, which could explain the decrease in the total m^6^A modification level. ALKBH5 may shorten the half-life of CYR61 mRNA in an m^6^A-dependent manner and impair the invasion ability of trophoblast cells [94]. Women who had spontaneous abortions had downregulated FTO, an RNA demethylase. Abnormal m^6^A accumulation at the maternal-foetal interface may therefore lead to spontaneous abortion (Fig. 2) [95].

### Endometriosis and adenomyosis

Endometriosis is an oestrogen-independent disorder defined as the presence of endometrium-like tissue outside the uterus, with patients suffering severe pelvic pain and infertility [96–98]. Although the aetiology of endometriosis remains unclear, its pathogenesis may also be linked to RNA methylation. For example, the first investigation into how m^6^A regulators function in endometriosis indicated that m^6^A regulators in endometriosis, such as METTL3, YTHDF2, YTHDF3, HNRNPC, HNRNPA2B1, and FTO, are significantly dysregulated compared with normal endometrial tissue [99]. This is consistent with a study that showed that the normal endometrium has higher levels of m^6^A than eutopic and ectopic endometrium [100]. The researchers also showed that the reduction in m^6^A in endometriosis was caused by METTL3 downregulation. Furthermore, they concluded that METTL3 downregulation inhibited the maturation of pri-miRNA126 to miRNA126 via DGCR8, thereby enhancing the migration and invasion of endometrial stromal cells and leading to the development of endometriosis. These results suggest that regulators of m^6^A RNA methylation, such as METTL3 or FTO, may become a potential marker for diagnosis and a treatment target for endometriosis.

As the name implies, adenomyosis is a uterine disorder in which the endometrium contains abnormally high levels of endometrial epithelial cells and stromal fibroblasts [101–104]. The myometrium of patients with adenomyosis showed lower levels of total m^6^A due to differential expression of m^6^A RNA methylation regulators, such as METTL3 and ZC3H13. In addition, they verified that the elevated gene expression of IGF1 and DDT was significantly correlated with decreased expression of METTL3. In a previous study, these genes were found to be involved in epithelial proliferation and cell migration. Adenomyosis may also be triggered by these processes [105, 106].

### Polycystic ovarian syndrome (PCOS)

Scientists have demonstrated that a putative m^6^A site within the 3'-UTR of FOXO3 mRNA mediates its mRNA decay by YTHDF2 in luteinized granulosa cells of controls [107]. It has already been demonstrated that FOXO3 may be involved in the aberrant autophagy, angiogenesis, and cell proliferation of sorafenib-resistant hepatocellular carcinoma in a METTL3-YTHDF1-dependent manner [108]. Interestingly, autophagy in ovarian tissue is very common in PCOS. Thus, the METTL3-FOXO3 axis may be associated with autophagy and insulin resistance in PCOS women [109]. Furthermore, granulosa cell dysfunctions such as proliferation, apoptosis, and autophagy are implicated in the pathogenesis of PCOS. By decreasing the m^6^A level of FLOT2 mRNA, FTO demethylates m^6^A, causing insulin resistance, high proliferation, and low apoptosis in granulosa cells [110]. A study on differentially expressed genes shared by PCOS and ovarian cancer (OC) showed that OGN, a small proteoglycan with tandem leucine-rich repeats (LRRs), was reduced in both PCOS and OC, and it can be inferred that low OGN expression is one of the important features of transformation from PCOS to OC. In this study, they confirmed that OGN expression in OC patients was positively correlated with the expression of several m^6^A regulators. We speculate that m^6^A can regulate the occurrence of OC and PCOS through a certain signalling pathway through OGN and even participate in the malignant transformation of PCOS [111].

### Primary ovarian insufficiency (POI)

Primary ovarian insufficiency (POI) is characterized by persistent amenorrhea, increased follicle-stimulating hormone levels, and low oestrogen levels. The patient was less than 40 years old and had amenorrhea for at least 4 months with increased follicle-stimulating hormone and low oestrogen levels. It is unclear how chemotherapy treatment causes premature ovarian failure in premenopausal women. Alkylating agents, such as CTX, are common causes of gonadal dysfunction [112–115]. Research proved that CTX increased the levels of m^6^A and its methyltransferases METTL3 and METTL14 as well as decreased the levels of its demethylase FTO and effector YTHDC1 in a concentration- and time-dependent manner in human granulosa cells and mouse models [116]. A previous study supported this conclusion, finding that the m^6^A level was elevated and FTO expression was decreased in POI patients. Additionally, a study showed that granulosa cells with FTO knockdown were significantly more likely to undergo apoptosis and to proliferate less [117]. Research on ALKBH5-deficient male mice found increased m^6^A in mRNA. The number of spermatozoa released by these mice was dramatically reduced; their morphology was abnormal, but their motility was altered. Additionally, mice deficient in ALKBH5 likely exhibited apoptosis of spermatocytes at the metaphase and pachytene stages, along with disordered spermiogenesis. The presence of ALKBH5 in both male and female reproductive tissues suggests that it may be vital to the development of germ cells [118]. Abnormal levels of these m^6^A regulators may contribute to impaired oocyte maturation by affecting cell division at the meiotic stage.

### m^6^A and female reproductive system tumours

Many previous studies have confirmed the role of RNA methylation in RNA metabolism. In addition, m^6^A may be involved in material and energy metabolism as a posttranscriptional modification. For instance, the Warburg effect, known to exist in tumour cells, tends to lyse glucose to lactate even when oxygen is sufficient to support mitochondrial oxidative phosphorylation. This is an essential feature of cancer cell metabolism. In the Warburg effect or aerobic glycolysis, hexokinase 2 plays a crucial role. METTL3 promotes cervical cancer cell proliferation and the Warburg effect by enhancing methylated hexokinase 2 mRNA stability through recruiting YTHDF1 [119]. PDK4 contributes to the production of ATP and glycolysis. IGF2BP3 and the YTHDF1/eEF-2 complex can sustain PDK4 stability through the m^6^A system, thus enhance glycolysis, and accelerates cervical carcinogenesis [120].

Cervical cancer is closely associated with human papillomavirus infection. A virus-derived circular RNA, circ E7, encodes the E7 oncoprotein. Its posttranscriptional modifications, such as m^6^A methylation, may play an important role in the expression of the HPV E7 oncoprotein, which promotes the transformation of cervical cells by HPV and the development of cervical cancer [121]. CENPK plays a proto-oncogene role in cervical carcinogenesis. In a study on the RNA methylation enzyme ZC3H13 promoting tumorigenesis in cervical cancer cells, m^6^A regulated CENPK mRNA methylation, which activates oncogenic function and drug resistance in cervical cancer cells through the ZC3H13-CENPK-SOX6-p53/Wnt regulatory axis [122]. In addition to mRNA m^6^A modification, circCCDC134 RNA methylation mediated by ALKBH5 and YTHDF2 is also involved in the proliferation and metastasis of cervical cancer cells [123]. FTO suppresses β-catenin transcription and enhances ERCC1 function in cervical squamous cell carcinoma, which decreases the cancer cells' susceptibility to chemotherapy [124].

By interacting with certain pathways, m^6^A regulators promote cell proliferation, migration, and invasion in some gynaecological malignancies. In endometrial cancer, FTO modulates the WNT signalling pathway, influencing the mRNA levels of HOXB13 and promoting tumour metastasis [125]. Mutations in m^6^A regulators can affect tumorigenesis. For example, hotspot R298P mutations occurring in the methylation enzyme METTL14 may be involved in the proliferation of endometrial cancer cells by affecting the activity of AKT pathways, such as the AKT regulators PHLPP2 and mTORC2 [126]. Another study found that the m^6^A reader IGF2BP1 enhanced PEG10 expression and promoted endometrial cancer cell proliferation by recognizing the m^6^A site in PEG10 mRNA [127]. Furthermore, high expression of IGF2BP1 was associated with poor prognosis in endometrial cancer patients. Another study on endometrial cancer also confirmed that m^6^A methylation levels were reduced in endometrial cancer cells and that m^6^A mRNA methylation could regulate the ERK/NF-κB/AKT signalling pathway through the PAPPA/IGFBP4 axis to induce proliferation and tumour formation in endometrial cancer cells [128]. In addition, it has been reported that IGF2BP1 is involved in the guanylation of arginine in endometrial cancer, and the PADI2/MEK1/ERK signalling pathway-mediated dysregulation of IGF2BP1 leads to the upregulation of SOX2 expression, resulting in the malignancy of endometrial cancer cells [129].

m^6^A RNA methylation regulators are associated with the malignancy and prognosis of ovarian cancer [130]. In a study on ovarian cancer, FBW7 counteracted the tumorigenic effect of YTHDF2 by inducing its proteasomal degradation [131]. In addition, researchers have demonstrated that the m^6^A reader YTHDF1 acts on EIF3C mRNA, a subunit of the protein translation initiation factor EIF3, to enhance protein translation efficiency in ovarian cancer cells in an m^6^A-dependent manner, which promotes ovarian carcinogenesis [132]. Meanwhile, high expression of YTHDF1 was associated with poor prognosis of ovarian cancer. IGF2BP1 promoted SRF expression in a conserved and m^6^A-dependent manner by impairing SRF mRNA attenuation. This phenomenon is closely associated with poor overall survival probability in ovarian cancer [133]. In contrast to the tumour-promoting effects of FTO reported in other studies, a study on ovarian cancer confirmed that FTO expression was suppressed in ovarian cancer and that the demethylation activity of FTO could inhibit the stemness characteristics and self-renewal of ovarian tumour stem cells by regulating the cAMP pathway [134]. Similar to the findings of this study, another study confirmed that low FTO expression was associated with higher FIGO staging in ovarian cancer patients and that FTO decreased the m^6^A level and stability of SNAI1 mRNA, leading to downregulation of SNAI1 expression and inhibition of the epithelial-mesenchymal transition [135].

Lymphatic metastasis plays a critical role in the progression of ovarian cancer cells. Studies have shown that ALKBH5 is overexpressed and m^6^A modification levels are reduced in ovarian cancer with lymph node metastasis and that the ALKBH5/m^6^A-ITGB1/FAK signalling axis plays an important role in ovarian cancer lymphangiogenesis and lymph node metastasis [136]. Targeted drugs against signalling molecules in this pathway may become an effective means to inhibit lymphatic metastasis in ovarian cancer. In another study on the tumour suppressor IFFO1, researchers found that the METTL3/YTHDF2 axis regulates the mRNA stability of IFFO1 in an m^6^A-dependent manner, which inhibits metastasis and reverses drug resistance in ovarian cancer cells [137]. By deactivating PI3K/AKT/mTOR, overexpression of METTL14 inhibited granulosa cell proliferation. The proliferation and migration of high-grade serous ovarian cancer cells were influenced by WTAP-mediated activation of the AKT signalling pathway [138]. It is interesting to note that METTL14 and WTAP knockdown slightly affected in vitro cell behaviour associated with tumour progression of endometrioid epithelial ovarian cancer cells [139]. This discrepancy may be attributed to a different type of pathology.

## Other RNA modifications in female reproductive system diseases

Based on the deepening of m^6^A modifications and transcriptome-wide mapping approaches, other posttranscriptional modifications of RNAs have also received attention, including N^1^-methyladenosine (m^1^A), 5-methylcytidine (m^5^C), N^7^-methylguanosine (m^7^G), and pseudouridine (Ψ) [140–142]. Although research on these chemical modifications is not as intensive as that on m^6^A, the role of these chemical modifications in female reproductive disorders deserves attention and has great therapeutic potential.

m^5^C is a conserved and common RNA modification. It is primarily found in eukaryotic tRNAs and rRNAs [143]. In an animal experiment, deletion of the m^5^C methyltransferase Nsun5 resulted in suppression of ovarian function and embryonic development arrest in mice [144]. In several bioinformatics studies on ovarian and endometrial cancers, m^5^C-related genes were closely associated with tumour chemotherapy sensitivity and overall survival [145–147]. In addition, abnormal m^5^C modification may be involved in TRDMT1-mediated granulosa cell death, which in turn causes premature ovarian failure [148]. Another study in *Drosophila* confirmed that YPS promotes the proliferation and differentiation of germinal stem cells in the *Drosophila* ovary by binding RNA containing m^5^C modifications [149]. However, the role of m^5^C in human ovarian function remains to be further investigated.

m^1^A and m^7^G modifications are also present in eukaryotic mRNAs [150]. m^1^A modifications may produce their biological effects by enhancing RNA‒protein interactions or by altering RNA secondary structures [151]. m^7^G modification is usually catalysed by METTL1 and located at the 5’ caps and internal positions of eukaryotic mRNA [152]. Several studies on endometrial cancer have found that m^1^A- and m^7^G-related lncRNAs and miRNAs can be used to construct tumour-related prognostic models and are associated with different immune infiltration phenotypes and drug susceptibility in endometrial cancer [153–156]. Pseudouridine is the most abundant modified nucleotide in RNA [157]. In a study on ovarian cancer, a pseudouridine synthase, PUS7, was considered a potential diagnostic marker for ovarian cancer [158].

## m^6^A RNA methylation sheds light on the diagnosis and treatment of several diseases

The TNM stage of gastric cancer is associated with METTL14 expression. According to an overall survival analysis, overall survival rates were higher for patients with higher levels of METTL14 expression. Based on multivariate analysis, METTL14 expression levels were associated with improved outcomes [159]. Non-small cell lung carcinoma cells undergo drug resistance and metastasis via diverse pathways when METTL3 increases m^6^A modification of both YAP and lncRNA MALAT1 [160]. FTO promotes cell proliferation of acute myeloid leukaemia in an m^6^A-dependent manner. Two inhibitors of FTO have been successfully developed by Huang and colleagues. These inhibitors, FB23 and FB23-2, bind directly to FTO and inhibit its m^6^A demethylase activity. The inhibitors display significantly improved inhibitory activity on FTO demethylation of m^6^A-RNA in vitro. Using these inhibitors, researchers inhibited the proliferation of a panel of AML cell lines and primary AML leukaemia stem cells in patient-derived xenotransplantation mice. Therefore, FTO and its analogues may be effective molecular targets for inhibiting leukaemogenesis in leukaemia stem cells [161–163]. Previous studies also indicated that YTHDF1 was strongly associated with a poor prognosis for ovarian cancer, breast cancer, and other female reproductive disorders [132, 164, 165]. The low expression of KIAA1429, an m^6^A methyltransferase, significantly increased dendritic cell infiltration. Researchers have shown that KIAA1429 expression affects immune checkpoint blockade therapeutic efficacy and boosts intratumoral antitumour immunity. Patients with low KIAA1429 expression experienced survival benefits from anti-PD-L1 immunotherapy [166–168].

It has been demonstrated that m^6^A regulators are associated with the prognosis of cervical cancer patients. m^6^A methylation regulators may be key regulators of PD-L1 expression and immune cell infiltration and may have an important impact on the TIME of cervical cancer [169]. In recent years, the role of m^6^A modification in PARP resistance in ovarian cancer has attracted the interest of several scientists. In BRCA-mutated ovarian cancer cells, m^6^A modification of FZD10 mRNA promoted PARP resistance through upregulation of the Wnt/β-catenin pathway [170]. Another study on olaparib resistance in ovarian cancer cells showed that m^6^A modifications of the olaparib pharmacogene were increased in resistant ovarian cancer cells [171]. These m^6^A modification sites could be used as potential pharmacoepitranscriptomics markers of drugs. Studies have confirmed that ALKBH5 expression is upregulated in cisplatin-resistant ovarian cancer and that overexpression of the ALKBH5-HOXA10 loop activates the JAK2/STAT3 signalling pathway, leading to chemoresistance in ovarian cancer [172]. In future studies, targeted blockade against the ALKBH5-HOXA10 loop may reverse chemoresistance in ovarian cancer cells. Based on the above studies, it is clear that m^6^A regulators are closely linked to clinical treatment and diagnosis.

## Conclusion

The actions of m^6^A modification have gradually become apparent in recent years as high-throughput sequencing technologies and highly specific antibodies against m^6^A have been developed. In the field of m^6^A RNA methylation, great progress has been made in revealing potential mechanisms of the onset and progression of female reproductive system disorders. Differentially expressed m^6^A regulator genes are found in a large number of gynaecological cells compared to normal cells and serve as triggers in gynaecological disease progression. Additionally, imbalanced RNA m^6^A has also been identified in these diseases. To clarify the molecular mechanisms underlying certain refractory obstetrics and gynaecology diseases, such as endometriosis and preeclampsia, abnormal RNA m^6^A modification is an important research direction.

Various female reproductive cancers are known to be affected by m^6^A modification and its regulators. Crystal structure data, for example, have provided insight into cancer-associated mutations in METTL14. Mutations in METTL14 that cause an inefficient RNA-binding domain are found in endometrial cancers, and the mutated form of the enzyme shows partially reduced activity. It has also been found that m^6^A mRNA methylation modulates AKT activity, which is necessary for endometrial cancer proliferation and tumorigenicity [126]. By binding to a locus of the MYC gene, IGF2BP2 enhances the proliferation, metastasis, and aerobic glycolysis of cervical cancer cells [173]. These findings indicate that the role of m^6^A regulators in female reproductive organs is different based on cell type, therapeutic issues, and pathological conditions.

Clinical diagnostics and therapeutics can also target m^6^A regulators in disorders of the female reproductive system. The oncogenic mechanism of mRNA m^6^A modification in gynaecologic malignant tumorigenesis may become a direction for future research. m^6^A regulators and their upstream and downstream signalling pathways may provide certain methods to elucidate the pathophysiological mechanisms of gynaecologic malignant tumours and establish prognostic models. Modification of pharmacogene mRNAs may alter their pharmacokinetics and pharmacodynamics, thus affecting drug efficacy. In future studies, not only do the pathophysiological mechanisms of female reproductive system diseases in which m^6^A is involved deserve attention but also the m^6^A modifications occurring in the pharmacodynamic gene targets corresponding to drugs need to be considered.

For the successful design of tissue- or cell-specific RNA methylation agonists or inhibitors, more multicentre and large-scale studies are needed. Many essential issues must be addressed in a detailed manner. A very limited number of cell and animal models are used in all cited papers, and the results are hence subject to variation. First, although METTL4, FTO, and ALKBH5 are commonly known and well-studied m^6^A regulatory factors, the role of less studied m^6^A regulatory factors, such as RBM15/15B and ZC3H13, may only be the tip of the iceberg. Further research is needed on the interactions between RNA modification regulators and their downstream targets in the female reproductive system. Second, the reproductive-endocrine system plays a pivotal role in the progression of gynaecological diseases. Research currently focuses on the influence of m^6^A on cellular behaviour. The interplay between the hormonal milieu and endocrine system related to m^6^A modification remains a mystery and should be addressed in the future. Finally, the majority of published studies have concentrated only on the molecular mechanism of m^6^A modification. A greater focus should be placed on the diagnostic and therapeutic properties of m^6^A. According to a large body of evidence, m^6^A modifications are related to clinical phenotypes and prognoses. m^6^A is involved in the common pathological features shared by benign and malignant diseases of the female reproductive system, and different m^6^A phenotypes in these diseases may be involved in the transformation between benign and malignant diseases and between different pathological types in the same disease.

Compared with other reviews in this field, our article provides a more comprehensive overview of the relationship between m^6^A and the physiology and pathology of the female reproductive system. Whether it is germ cell genesis, embryonic development or benign and malignant diseases of the female reproductive system, we searched for appropriate literature to address them. In particular, we briefly outlined other RNA modifications. The connection between these RNA modifications and m^6^A modifications may become a new direction in future studies. In addition, we look forward to the involvement of m^6^A modifications in the diagnosis and treatment of female reproductive system diseases. m^6^A modifications deserve to be noticed for their clinical applications in female reproductive system diseases.

Undoubtedly, the pathogenesis of female reproductive system diseases is quite complex, and changes in the levels of m^6^A regulators dynamically regulate the level of m^6^A modifications in the female reproductive system, altering the abundance and function of related mRNAs and proteins and ultimately determining the onset of disease. However, the application of m^6^A regulators to clinical diagnosis and treatment requires improving the sensitivity and specificity of the relevant biomarkers. RNA m^6^A modification can likely be used to classify clinical phenotypes of several female reproductive disorders and to predict the mutual transformation of these disorders.

## Data Availability

Not applicable.

## Abbreviations

3′ UTR: 3'-Untranslated region
ALCAM: Activated leukocyte cell adhesion molecule
APA: Alternative polyadenylation
BAMBI: BMP and activin membrane bound inhibitor
BPDE: Benzo(a)pyrene-trans-7,8-diol-9,10-epoxide
CDS: Sequence coding for aminoacids in protein
CNOT1: CCR4-NOT transcription complex subunit 1
CRD: Coding region instability determinant
CREBBP: CREB binding protein
CTX: Cytoxan
CYR61: Cysteine-rich 61
DDT: D-dopachrome tautomerase
DGCR8: DiGeorge syndrome critical region gene 8
FBXW7: F-box and WD repeat-containing 7
FOXO3: Forkhead box O3;
GV: Germinal vesicle
hnRNP: Heterogeneous nuclear ribonucleoprotein
HOXB13: Homeobox B13
HSPA1: Heat Shock Protein 70
IGF1: Insulin-like growth factor-1
IGF2BP: Insulin-like growth factor 2 mRNA-binding protein
ITSN2: Intersectin 2
KDM3B: Lysine demethylase 3B
VIRMA: Vir like m^6^A methyltransferase associated
lncRNA: Long non-coding RNA
LRR: Leucine-rich repeat
m^6^A: N^6^-methyladenosine
m^6^Am: N6,2′-O-dimethyladenosine
MAT2A: Methionine adenosyltransferase 2A
METTL14: Methyltransferase-like Protein 14
MII: Metaphase II
MTC: N^6^-adenosine methyltransferase complex
MXD1: MAX dimerization protein 1
MYB: MYB proto-oncogene, transcription factor
MYC: MYC proto-oncogene
NRL: Nucleosomal repeat length
NXF1: Nuclear RNA export factor 1
OC: Ovarian cancer
OGN: Osteoglycin
PAPPA2: Pappalysin 2
PD-L1: Programmed cell death-ligand 1
PPARG: Peroxisome proliferator activated receptor gamma
pre-mRNA: Pre-messenger RNA
RBM15/15B: RNA binding motif protein 15/15B
SAM: S-adenosylmethionine
SMPD1: Sphingomyelin phosphodiesterase 1
SRSF3: Serine and arginine rich splicing factor 3
TNM: Tumor node metastasis
U6 snRNA: U6 spliceosomal small nuclear RNA
VCR: Vertebrate conserved region
WTAP: Wilms tumor 1-associating protein
XIST: X-inactive specific transcript
YTH family: YT521-B homology domain family
ZC3H13: Zinc finger CCCH-type containing 13
